# P-2175. A Pilot Study to Establish a Nursing Home Syndromic Surveillance Network

**DOI:** 10.1093/ofid/ofaf695.2338

**Published:** 2026-01-11

**Authors:** H Edward Davidson, Lisa Han, Kevin McConeghy, Yasin Abul, Ivis Perez, Evan Dickerson, Laurel Holland, Tiffany Wallace, Mandi Winkis, Clare Nugent, Olajide Olagunju, Debbie Keresztesy, Elise Didion, David Canaday, Stefan Gravenstein

**Affiliations:** Insight Therapeutics LLC, Norfolk, Virginia; Insight Therapeutics, LLC, Norfolk, Virginia; COIN-LTSS, Providence Veterans Affairs Medical Center, Providence, Rhode Island; Brown University, Providence, Rhode Island; Insight Theraputics, Norfolk, Virginia; Brown University, Providence, Rhode Island; Rhode Island Hospital, Providence, Rhode Island; Brown University Health, Providence, Rhode Island; Rhode Island Hospital, Providence, Rhode Island; Rhode Island Hospital, Providence, Rhode Island; Case Western Reserve University, Cleveland, Ohio; CWRU, cleveland, Ohio; Case Western Reserve University, Cleveland, Ohio; VA Northeast Ohio Healthcare System, Cleveland, OH; Brown University, Providence, Rhode Island

## Abstract

**Background:**

Timely local surveillance data regarding acute respiratory infections (ARI) is crucial for nursing homes (NHs) to proactively manage outbreaks. Centers for Disease Control and Prevention (CDC) national-level viral surveillance data lags by weeks, hindering timely and informed decisions. Our study evaluated feasibility and utility of a pragmatic approach to improve respiratory virus testing.

**Figure:**
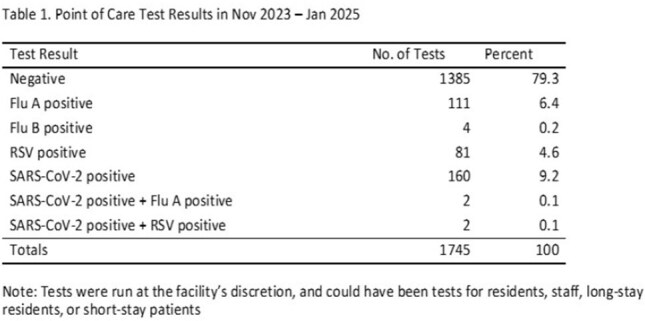
Point of Care Test Results in Nov 2023 – Jan 2025

**Methods:**

We enrolled 23 NHs in OH, MO, IN, RI, VA, and PA. We placed a CLIA-waived Cepheid GeneXpert Xpress point-of-care (POC) device, to enable rapid (< 30 minutes) on-site molecular testing for Respiratory Syncytial Virus (RSV), Severe Acute Respiratory Syndrome Coronavirus 2 (SARS-CoV-2), and influenza A and B from long- and short-stay residents’ and staff members’ nasal swabs. The device enabled reported symptoms to be entered at the time of testing and automatically transmitted results to a web-based dashboard, providing real-time feedback on respiratory viral activity from Nov 2023 — Jan 2025. Monthly surveys of NH staff gathered qualitative feedback on use of the device, impact on testing protocols, and virus awareness.

**Results:**

Enrolled NHs had a mean bed size of 155 (range 50-427) averaging a monthly census of 125 residents. Of 1745 POC tests, 360 (20.6%) identified a virus: 111 (6.4%) influenza A, 4 (0.2%) influenza B, 81 (4.6%) RSV, and 160 (9.2%) SARS-CoV-2; 4 individuals with coinfection were also identified (Table 1).

Barriers for initial setup included: unfamiliar technology, device placement issues, staff training, network access, need for additional data entry by busy NH staff, resistance to change, and staff turnover. Monthly surveys revealed that the presence of the device impacted testing protocols, led to a greater recognition of respiratory virus infections among staff, and reduced time to diagnose these infections.

**Conclusion:**

We demonstrated the feasibility and utility of implementing CLIA-waived POC multiplex molecular testing for viral ARIs within a network of nursing homes. The high test-positivity (20.6%) demonstrated value for clinical management and an approach that could be adapted for regional or national real-time reporting.

**Disclosures:**

H Edward Davidson, PharmD, GSK: Grant/Research Support|Moderna: Grant/Research Support|Sumitomo: Grant/Research Support Lisa Han, MPH, GSK: Grant/Research Support|Moderna: Grant/Research Support|Sumitomo: Grant/Research Support Kevin McConeghy, Pharm.D., GlaxoSmithKline: Investigator-Initiated Study|Moderna: Grant/Research Support Yasin Abul, MD, CDC/ABT: Grant/Research Support|CLARIO: Advisor/Consultant|GSK: Grant/Research Support|Moderna: Grant/Research Support|Seqirus: Grant/Research Support Ivis Perez, MPH, LPN, GSK: Grant/Research Support|Moderna: Grant/Research Support|Sumitomo: Grant/Research Support David Canaday, MD, Moderna: Grant/Research Support|Pfizer: Grant/Research Support|Seqirus: Advisor/Consultant|Seqirus: Grant/Research Support|Seqirus: Honoraria Stefan Gravenstein, MD, MPH, GSK: Advisor/Consultant|GSK: Grant/Research Support|GSK: Honoraria|Moderna: Grant/Research Support|Novavax: Advisor/Consultant|Novavax: Honoraria|Pfizer: Advisor/Consultant|Pfizer: Grant/Research Support|Pfizer: Honoraria|Sanofi: Advisor/Consultant|Sanofi: Grant/Research Support|Sanofi: Honoraria|Seqirus: Grant/Research Support

